# Development of tensile strength methodology for murine skin wound healing

**DOI:** 10.1016/j.mex.2018.04.002

**Published:** 2018-04-16

**Authors:** Anuj Bellare, Michael W. Epperly, Joel S. Greenberger, Renee Fisher, Julie Glowacki

**Affiliations:** a Department of Orthopedic Surgery, Brigham & Women’s Hospital, Harvard Medical School, Boston, MA, USA; b Department of Radiation Oncology, University of Pittsburgh Cancer Institute, Pittsburgh, PA, USA

**Keywords:** Tensile testing for murine skin wound, Tensile strength, Wound repair, Murine skin, Biomechanical test

## Abstract

In this study, a methodology was evaluated and improved to quickly measure the tensile strength of murine skin in a biomechanical assay for an incisional wound healing model. The aim was to streamline and enhance the wound model, skin specimen preparation, and tensile test so that large numbers of fresh tissue could be tested reliably and rapidly. Linear incisions of 25-mm length were made in the dorsal skin of mice along the spine and metallic staples were used to close the wound. After 20 days, the mice were sacrificed, and a square-shaped section of skin containing the linear incision was excised. Two metallic punches were fabricated and used to punch 15-mm long strips of skin of 2 mm width whose length was orthogonal to the direction of incision. The tensiometer configuration was modified to expedite tensile measurements on fresh skin, and load-to-failure was measured for each strip of skin from the cephalad to the caudal region. We evaluated sources of error in the animal model and the testing protocol and developed procedures to maximize speed and reproducibility in tensile strength measurements. This report provides guidance for efficient and reproducible tensile strength measurement of large numbers of skin specimens from freshly sacrificed animals.

• Tattoo placement to identify the two ends of the healing incisional wound assisted in decreasing error in the position and orientation of tensile strips.
• Custom-made punches to prepare skin strips for tensile testing helped conduct tensile tests of fresh tissue rapidly.
• Alteration of the manual grips of the tensile tester enabled specimens to be gripped rapidly to significantly accelerate testing for each skin strip.

## Graphical abstract

**Figure:**
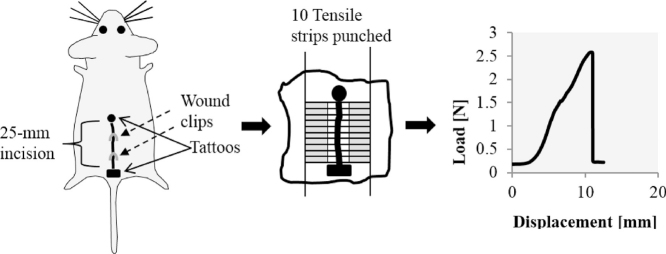

Specifications Table

**Table:** 

Subject area	*Select one of the following subject areas:*
	• *Medicine and Dentistry*
More specific subject area	*Healing of skin wound*
Method name	*Tensile Testing for Murine Skin Wound*
Name and reference of original method	Gorodetsky, R., W.H. McBride, and H.R. Withers, *ASSAY OF RADIATION EFFECTS IN MOUSE SKIN AS EXPRESSED IN WOUND-HEALING.* Radiation Research, 1988. **116**(1): p. 135-144.
	Gorodetsky, R., et al., *EFFECT OF FIBROBLAST IMPLANTS ON WOUND-HEALING OF IRRADIATED SKIN - ASSAY OF WOUND STRENGTH AND QUANTITATIVE IMMUNOHISTOLOGY OF COLLAGEN.* Radiation Research, 1991. **125**(2): p. 181-186.
	Gorodetsky, R., et al., *RADIATION EFFECT IN MOUSE SKIN - DOSE FRACTIONATION AND WOUND-HEALING.* International Journal of Radiation Oncology Biology Physics, 1990. **18**(5): p. 1077-1081.
Resource availability	*N/A*

## Methods detail

Our understanding of the complexity of skin wound healing has increased with the use of various, highly reproducible animal models that can address specific questions with quantitative outcome measures, especially the restoration of biomechanical properties. These complex processes involving inflammatory, fibrobastic, and maturation phases of healing have been extensively investigated in order to develop drugs and devices to treat wounds so that the post-wound skin remodeling results in a morphology that closely resembles that of the intact skin [[1], [2], [3], [4], [5], [6]]. Biomechanical assays serve as useful techniques to determine the extent of skin wound healing and provide insight into the functionality of repaired skin compared with intact skin. Several types of biomechanical assays have been developed that measure the static tensile [[7], [8], [9], [10], [11], [12], [13], [14], [15]], biaxial tensile [16], and rheological properties [[17], [18], [19]] of skin to assess wound healing, aging, drug therapies, and biomaterials used for wound dressings. Among these biomechanical assays, the most common and simplest assay is the tensile test. This test often measures the tensile failure load or stress or the modulus (stiffness) of skin under different conditions. In the absence of standardized tensile test protocols, various investigators use different test specimen geometries and tensile stretching rates to measure tensile properties of skin. These test specimen geometries often depend on the animal model used and the region of the body of the skin being excised.

This study concerns skin incisions with primary closure to serve as an experimental model for controlled clinical surgical settings. This report concerns modifications made to expedite handling a large number of specimens to measure healing of incisional wounds in mice. Pilot studies revealed a lack of feasibility to process large numbers of samples with a standard tensiometer. To solve this limitation, elements of both a mouse skin injury model and of a protocol for measurement of tensile strength were modified.

## Materials and methods

### Micro-tensiometer

An eXpert 4000 micro-tester (Admet Inc, Norwood MA) with a frame capacity of 45 N and a spatial resolution of 0.0046 mm was used to measure tensile strength of strips of murine skin. The micro-tester was equipped with a 10 N and 50 N load cell and capable of crosshead speeds of up to 500 mm/min and a maximum displacement of 25-mm. The 10 N load cell was used for these studies. The tensiometer has two serrated manual screw-action clamps, each with two screws to secure specimens during tensile deformation. In order to decrease the time required to mount and dismount specimens, the screws of the clamps were set aside and two other clamping approaches were tested: we tried office Bulldog clamps and corrosion-resistant miniature spring clamps (McMaster-Carr, Robbinsville, NJ) to secure the specimens. MTEST Quattro Controller for the micro-tensiometer enables both static as well as cyclic testing in both tension and compression modes; these tests were conducted in static mode, according to the test conditions described by Gorodetsky [[8], [9], [10]].

### Materials for custom-built skin punches

We constructed two skin punches to expedite the reproducible preparation of skin strips. The first punch was designed to section an excised, approximately 25-mm × 25-mm segment of mouse dorsal skin into a precise 15-mm wide, 25-mm long rectangle; the second was designed to section 10 skin specimens of 2-mm width and 15-mm length from the skin rectangle. The following materials were used: (1) clear polycarbonate bar (McMaster-Carr, Princeton, NJ), (2) tight-tolerance 6061 Aluminum bar of 1.75-mm thickness and 3-mm width (McMaster-Carr, Princeton, NJ), (3) Tissue Path™ High-Profile microtome blades of 0.25-mm thickness and 50-mm length (Fisher Scientific, Waltham, MA), and (4) machinable epoxy casting compound Shore 86D (McMaster-Carr, Princeton, NJ).

### Preparation of punches

**Fig. 1:**
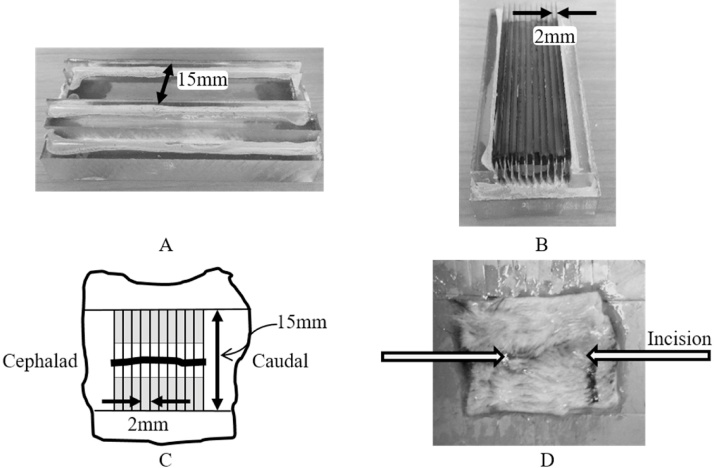
Custom-built punches used (A) to prepare rectangle of skin centered on the incision and (B) to prepare ten 15-mm long, 2-mm wide strips of skin for tensile testing. (C) A schematic of the orientation of tensile strips using the two punches in succession (shaded region represents the portion of specimens that would be gripped). (D) A representative skin specimen. The ends of the arrows approximate the location of the ends of the incisional wound.

Two punches, a 2-blade and an 11-blade one, were constructed to ensure that uniform strips of skin were prepared for tensile strength measurements with accurate dimensions. Initial manual attempts with scalpel blades to section strips introduced errors in the width, linearity, and orientation of the specimens with respect to the wound. While multi-blade slicers can also be used to prepare skin strips of fixed width, but we preferred to construct punches to avoid the skin strips being potentially overstretched during the slicing process. For the 2-blade punch, a 15-mm wide aluminum spacer was placed between the two blades along with a 3-mm thick spacer on the outer surfaces of the blades to maintain the blades in an upright position (Fig. 1A). This construct was held together with a rubber band and placed on a clear Polycarbonate slab, which served as a handle. The blades were carefully aligned to the slab and a wooden applicator was used to spread the epoxy compound on all contacting surfaces. The construct was then placed in an oven at a temperature of 60 °C overnight to cure the epoxy. A similar approach was used to prepare an 11-blade punch. Spacers of 1.75-mm thickness were carefully placed between each of 11 blades of 0.25-mm thickness to provide skin sections of 2-mm width. The first and eleventh blade had an outer spacer of 3-mm thickness to ensure that they would remain upright during the curing process. The entire construct was held together with a rubber band and placed on a slab of Polycarbonate. The epoxy was cured overnight at 60 °C and the hardened, set 11-blade punch was ready for use within 24 h (Fig. 1B). These punches could be used in succession, with alcohol rinses between specimens, to prepare tensile skin strips of precise length and width with respect to the wound, as depicted in Fig. 1C.

### Animal model

All animal protocols were approved by the University of Pittsburgh Institutional Animal Care and Use Committee. The University of Pittsburgh animal facility has been approved by the American Association of Laboratory Animal Care (AALAC). The mice (C57BL/6NTac female, Harlan Sprague Dawley, Chicago, IL) were anesthetized with isoflurane and fur was removed with an electric shaver, followed by depilation with NAIR®. In 5 experimental mice, scalpel was used to make a 25-mm incisional wound in the lower midline dorsal skin, and the incision was closed with two staples. For 48 h after surgery, the mice were injected twice a day with 0.1 mg/kg of buprenorphine hydrochloride. No wound dressing was used to treat the mice other than closing the wound with wound clips. The mice were housed 5 per cage and daily observations by our technicians and veterinary staff showed no evidence of scratching to the wounds of these mice. For removal of any staples that remained on day 7, the mice were anesthetized with isoflurane and staples removed with a wound clip remover. Groups of five experimental and five control, unwounded mice were sacrificed on day 20 by inhalation of carbon dioxide followed by cervical dislocation. Fur was removed from the backs of with an electric shaver, followed by depilation with NAIR®. An approximately 25-mm × 25-mm square shaped skin specimen was removed with scissors such that the wound was parallel to one length of the specimen and centered between the two edges (Fig. 1D).

### Preparation of skin strips for tensile strength measurements

The square shaped skin specimens were placed in a Petri dish containing phosphate buffered saline (PBS). The skin was transferred onto a 3-mm thick rubber sheet placed on a large polyethylene block. The custom-built 2-blade punch with a blade spacing of 15-mm was placed over the skin specimen and a ruler was used to align the punch so that the wound was close to the center between the two edges being sectioned. The punch was then manually pressed down until the blades penetrated the rubber sheet ensuring that the skin specimen had been sectioned completely. Thereafter, the 11-blade punch with spacings of 2-mm between blades was aligned orthogonal to the incision and placed on top of the skin specimen carefully so that the punch covered the entire specimen. The punch was pressed down manually to provide 10 strips of 2-mm width and 15-mm length. The strips were transferred to a Petri dish containing PBS for tensile testing while maintaining the cephalad-caudal orientation of each strip. Thereafter, all tensile strips were subjected to tensile testing until failure.

## Results

### Increasing the rate of measuring tensile strength of skin strips

**Fig. 2:**
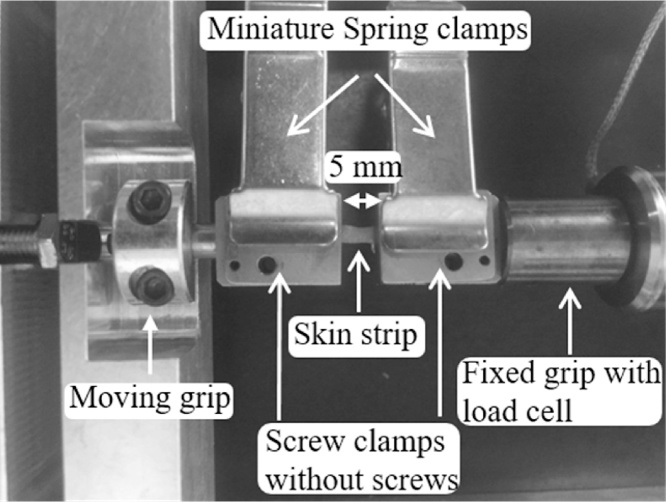
Tensiometer set-up with mouse skin mounted for tensile deformation using spring clamps.

The tensiometer was positioned horizontally on a benchtop so that the direction of extension was in a horizontal direction, an orientation that also facilitated specimen mounting. In a typical test, the two clamps of the tensiometer were maintained 5-mm apart with a 5-mm spacer and zeroed for both position and load. Zeroing was performed at hourly intervals to ensure no drift in load or displacement over time. Initial tests with the specimen clamps that came with the tensiometer revealed that securing each strip with the four screws consumed too much time, with risk of strip dehydration. First, we replaced the screw-on clamps with office Bulldog clamps with some success. Ultimately, stainless steel spring clamps were used to secure the skin strip to the tensiometer (Fig. 2). This modification ensured that all specimens would be gripped with the same clamping force. Second, it ensured that the applied clamping force would not tear the skin prior to the start of the test whereas manual tightening of the screws secure enough to prevent slippage could inadvertently damage or tear the skin specimen. Third, it saved approximately 4–7 min for mounting and dismounting each specimen. We ensured that the specimens remained slack after clamping so that there would be no applied load prior to the starting time of the measurement. After the test began, the sample was stretched to its original length and become taut, after which the specimen would be subjected to a tensile load.

**Fig. 3:**
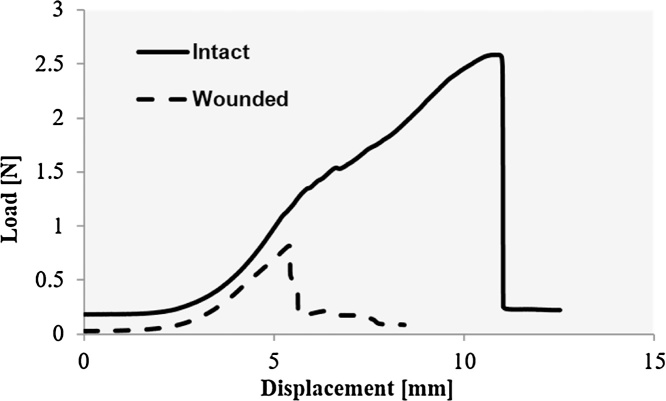
Representative load-displacement curve of a control, intact skin strip and a skin strip from an incisional wound at 20 days after incision.

Tensile tests were conducted at a crosshead speed of 25 mm/min; most wound specimens failed within 30 s of tensile deformation, corresponding to a maximum elongation of 12.5 mm (Fig. 3). The crosshead speed of 25 mm/min was chosen based on previous studies conducted to measure wound strength [8−10]. Such a high rate of deformation also allowed a large number of specimens to be measured in a short time duration, which would enable quantitative and statistically significant comparison of wound strength during healing under different conditions. In general, the duration of mounting and dismounting the specimen exceeded the duration of testing. For example, the duration of testing 10 specimens when screw clamps were used was approximately 1–1.5 h, whereas the use of spring clamps enabled all 10 specimens to be tested in approximately 20 min. This modification ensured that the tensile strength measurement of skin represented that of relatively fresh skin specimens compared to the specimens tested using clamps that were secured with 4 screws.

### Effect of spatial distribution on tensile strength of wounded and intact skin

The tensile strength of intact skin was 2.3 ± 0.1 N (mean ± standard error) and that of wounded skin after 20 days of healing was 1.4 ± 0.2 N. We chose a 20-day healing time point for the C57BL/6NTac female mice based upon Gorodetsky’s data with C3Hf/Sed/Kam mice genotype [8].The results, showing an almost 40% lower wound strength after 20 days post-incision (1.4 ± 0.2 N) compared with intact skin (2.3 ± 0.1 N) confirm that wound healing rates for these mice were slow. A different timeline may be appropriate for another animal model and/or different types of wounds.

**Fig. 4:**
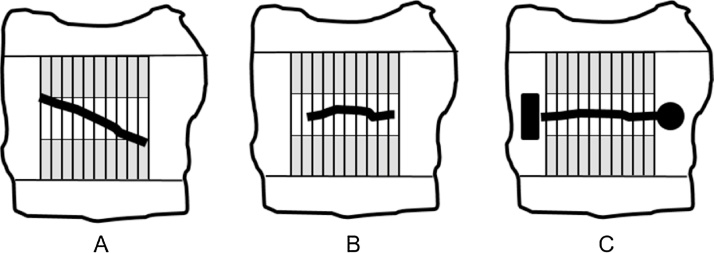
Schematic showing (A) a skin specimen with misalignment of incision with respect to tensile strips, (B) a skin specimen in which the incision is shorter than total width of 10 strips, and (C) a skin specimen with an incision between two different tattoo marks to indicate cephalad and caudal edges of the wound (shaded regions represent the region of the skin strips that are gripped by the clamps).

There was no discernible trend in the tensile strength of strips from the cephalad to the caudal region of the intact, control skin (Fig. 4 Table). Some values of tensile strength for the first and last strips at the opposite ends of the wound, however, showed values higher than the central specimens.

## Discussion

The report summarizes modifications we made to a mouse skin incisional wound and to a protocol for tensile testing to ensure reproducibility and handling of large numbers of specimens. We recommend tattooing the ends of the skin incision to retain orientation of ten skin strips perpendicular to the incision and with respect to cephalad-to-caudal direction of strips; this canprevent inadvertent inclusion of intact skin beyond the ends of the incision. We built two skin punches to make uniform skin strips for tensile testing. We found it necessary to modify the way the skin strip was secured in the tensiometer. The standard gripping devices outfitted by the tensiometer manufacturer have four screws to secure each strip, but screw adjustment is time-consuming and has the risk of specimen-to-specimen variations in clamping force and of tearing from excessive clamping force. Use of inexpensive spring clamps avoided those limitations. Please note that an alternative to spring clamps would be to use pneumatic clamps, which can also be used to rapidly clamp specimens and the pressure can be pre-adjusted to ensure that the skin strips would not tear under the clamping force.

The measured value of tensile strength of intact skin of 2.3 ± 0.1 N is relatively close to the range of tensile strength measured previously by Gorodetsky et al. who observed a tensile strength range of approximately 260 g – 400 g (2.5 N – 3.9 N) using similar specimen geometry and test conditions [8]. Bermudez et al. observed a tensile strength of intact skin of C57BKS female mice of 2.1 ± 0.5 MPA (4.2 ± 1.0 N) [20]. In another study, the intact dorsal skin of wild type mice displayed a tensile strength of 2.9 ± 0.8 N [21]. Small differences in tensile strength may be attributed to different genotypes of mice used in the respective studies and/or different tensile test conditions. The tensile strength of the skin with incisional wound after 20 days of healing also compare favorably with the study of Gorodetsky et al. who measured a tensile strength of approximately 1.25 g (1.2 N) while the corresponding tensile strength of our study was 1.4 ± 0.2 N.

The higher values of tensile strength observed for some strips from the ends of the wound (Table 1) may be due to regional healing occurring from the edge of the incision into the central regions of the wound. It is also possible, however, that the higher values were due to alignment errors in the experiment. For example, after the wound heals to a sufficient extent, it is not clearly visible, making it difficult to excise the skin specimen in accurate alignment to the wound. It is also difficult to cut the tensile strips with the incision perfectly aligned with the edges and centered on the tensile strips if the excised skin is not perfectly square shaped, which is difficult to do with scissors. Misalignment of the wound or a short incision can result in the tensile strips at the outer edges being stretched with intact skin in the test regions of the specimen (Fig. 4A and B). In order to avoid such alignment errors, it is therefore recommended that tattoos be placed at the outer ends of the incision, so that during healing as the incision becomes less visible, it would remain possible to align the test strips accurately using the tattoo marks as a reference (Fig. 4C). It is preferable for the two tattoo marks to differ in shape or size to distinguish between the cephalad and caudal regions associated with the excised skin specimen. It is also preferable for the incision length be measured carefully so that all specimens have incisions of identical length.

**Table 1:** Tensile Strength Measurements of Ten Strips from Intact skin and Skin with an Incisional Wound (10 strips per mouse and 5 mice per group). The tensile strength values of strips are arranged left to right in sequence from the cephalad to the caudal region. *indicates strips at the “ends” of the incision with values greater than for strips “within” the incisions.

	Mouse	Tensile Strength [N]									
		#1	#2	#3	#4	#5	#6	#7	#8	#9	#10
Intact skin	1	1.2	2.7	2.4	2.1	2.6	2.6	2.6	2.5	2.3	3.1
	2	2.2	2.2	2.6	2.6	2.1	1.8	2.1	2	1.6	1.8
	3	2.9	3	3	1.9	2.3	2	2.3	2.5	2.8	2.7
	4	3.2	3.2	2.8	2.6	2.5	2.1	2.1	2.1	2	2
	5	2.5	2.2	2.4	1.9	2.1	2.1	1.8	2.1	2.3	2.2
Wounded skin	1	1.9*	1.2	1.3	1.0	1.0	0.7	0.9	0.7	1.0	1.3
	2	2.8*	2.0	1.4	1.6	1.3	1.3	1.2	1.3	0.9	1.4
	3	2.2	2.2	1.6	0.9	0.6	0.9	0.9	1.5	0.9	1.9
	4	3.2*	2.9	2.6	1.1	1.1	0.8	0.9	1.2	0.8	1.4
	5	2.1*	1.4	1.6	0.9	0.9	1.0	1.1	1.2	0.9	3.2*

This study concerned skin incisional wounds with primary closure to serve as a model for controlled clinical surgical settings. Many other models have been developed for other research questions [22], for example, incisional wounds left open to heal by secondary intention. Excisional wounds entail the removal of a significant volume of skin without reduction of the tissue gap. In those cases, a clot is followed by granulation tissue, gradual re-epithelialization, and, in small, loose-skinned animals, closure by contraction of the margins. Wound healing models have been developed for many species, notably pig, rabbit, rats, and mice [22], with growing numbers of transgenic models in pigs and mice [23]. Tensile strength of healing wounds is usually determined by measuring the point of rupture of a segment of the wound on a tensiometer loaded at a fixed rate. Circular excisional models are used very frequently because of similarity to important chronic, non-healing human wounds such as venous leg ulcers and diabetic foot ulcers [24]
